# A new method to determine the QRS axis—QRS axis determination

**DOI:** 10.1002/clc.23477

**Published:** 2020-10-06

**Authors:** Qijun Gao, Zhiguo Dai, Yingfu Hu, Fang Bie, Bo Yang

**Affiliations:** ^1^ Department of Cardiology The First People's Hospital of Jingmen Jingmen Hubei Province PR China; ^2^ Department of Cardiology Renmin Hospital of Wuhan University Wuhan Hubei Province PR China

**Keywords:** mean QRS axis, new method, P‐ÂQRS

## Abstract

**Background:**

The development of a perfect method for determining the mean QRS axis (ÂQRS) is still lacking.

**Hypothesis:**

We proposed a new simple method, whether this method is accurate is unknown.

**Methods:**

The axis perpendicular to the mean QRS axis (P‐ÂQRS) divides six limb leads into two groups. All the leads that are in the range of 180° along the ÂQRS are positive, while all the leads in another 180° are negative, one lead is isodiphasic if it is on the P‐ÂQRS. If no lead is isodiphasic, then the P‐ÂQRS is located in the middle of two adjacent leads, which can help us determine the P‐ÂQRS. The six limb leads that fall in the range of −30° to 120° are as follows: aVL (−30°), I (0°), −aVR (30°), II (60°), aVF (90°), and III (120°). We can check an external lead (aVL or III) first. For example, if lead III is isodiphasic and lead aVF is positive, the P‐ÂQRS is 120°; if lead III is negative and lead aVF is positive, then the P‐ÂQRS is 105°. If more than one lead is negative, all such leads can be checked individually until a positive or isodiphasic lead is found. The ÂQRS can be easily decided once we know the P‐ÂQRS. In total, 200 recorded ECGs were investigated. We obtained the ÂQRS from our new method, computer interpretations, and a standard bipolar method. The Pearson correlation coefficient and Bland‐Altman analysis were performed.

**Results:**

The mean and SDs were remarkably similar, the correlation coefficient between the P‐ÂQRS method and the bipolar method was 0.976 (*P* < .001). Mean bias (Bland‐Altman limits of agreement) between the two methods was 0.885 (−12.37 to 14.14).

**Conclusion:**

The new method is simple and is able to assess the mean QRS axis accurately.

## 1 INTRODUCTION

Electrocardiograms (ECGs) are the recording of cardiac electrical activity. The axis of an ECG is the major direction of the overall electrical activity of the heart, which can be determined by analyzing the magnitude and size of the QRS complex of the limb leads. Existing methods for determining the mean QRS axis (ÂQRS) are either complicated requiring calculation, or simple but lack of accuracy.^1^ Some convenient methods have been provided in recent years, but they are still too complicated to be widely used.^2^ In a clinical context, if only the approximate range of ÂQRS is required, the ÂQRS can be assessed by leads I and aVF. If both leads are positive, the ÂQRS is normal (from 0° to 90°). The method of determining the electrocardiogram axis by isodiphasic lead (isodiphasic lead method) has been popular for half a century, since Grant raised it.^3^ In this method, the ÂQRS is perpendicular to the isodiphasic lead. However, this method cannot be used if none of the leads exhibits an isodiphasic lead. We have optimized this method and made it suitable for all electrocardiographs.

## 2 METHODS

**FIGURE 1:**
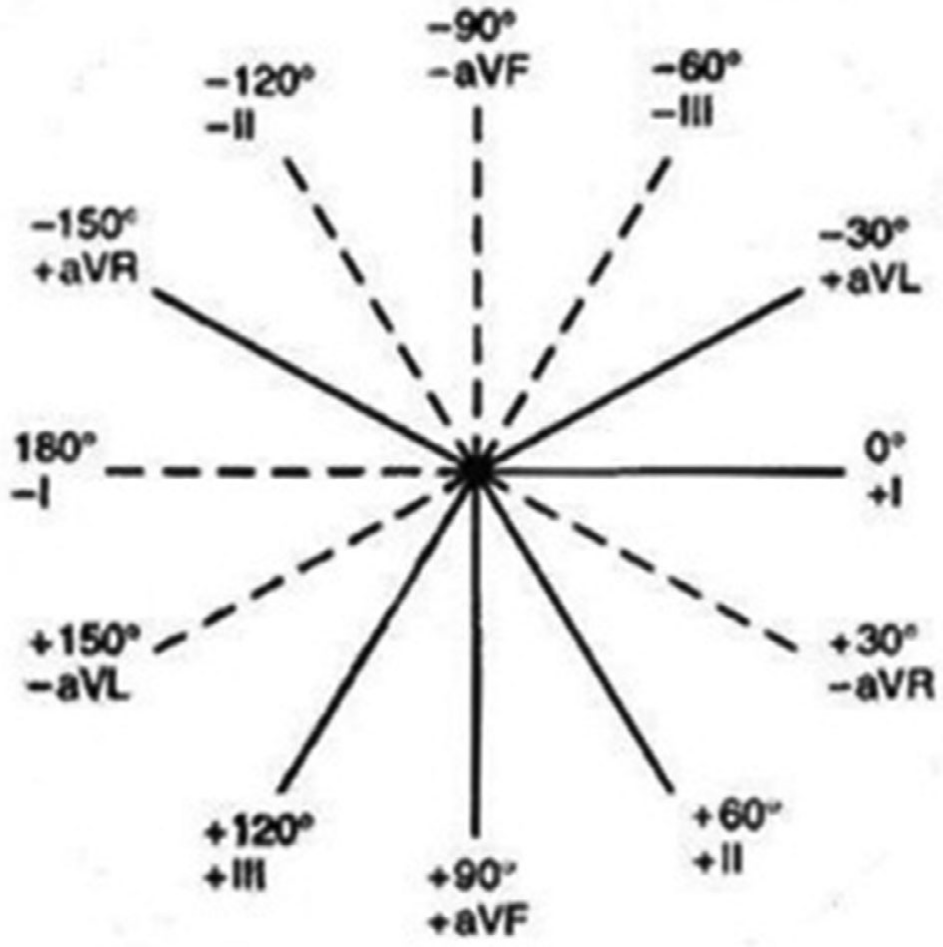
The hexaxial system

The axis perpendicular to the mean QRS axis (P‐ÂQRS) divides six limb leads into two groups. All the leads in a 180° range along the ÂQRS are positive, while all the leads in a 180° range against the ÂQRS are negative, one lead is isodiphasic if it is on the P‐ÂQRS. If no lead is isodiphasic, then the P‐ÂQRS is located in the middle of two adjacent leads. The six limb leads that fall in the range of −30° to 120° are as follows (referred to as the Cabrera sequence^4^): aVL (−30°), I (0°), −aVR (30°), II (60°), aVF (90°), and III (120°) (Figure 1). When the ÂQRS is at +45°, the morphology is positive in all the leads (aVR is negative, meaning that −aVR is positive), because the main vector of the mean QRS axis falls within the positive hemifield of all leads (with the exception of aVR). When the ÂQRS shifts to the left from +45° to +30° and as far as −120°, the P‐ÂQRS shifts from 135 to 130. Up to −30, the QRS complexes become negative, starting with lead III, changing from positive to isodiphasic and then from isodiphasic to negative for each shift in the ÂQRS of 15° left (Table 1A). As the ÂQRS shifts to the right from +45° to +60° and up to 180°, the complexes again become negative, but starting with lead aVL, they change from positive to isodiphasic and then from isodiphasic to negative with every 15° shift to the right in the electrical axis (Table 1B). When the mean QRS axis changes, the morphology of leads around the P‐ÂQRS changes; this allows us to easily determine the P‐ÂQRS from the morphology of limb leads. In most cases, the majority of leads are positive, making it easier to identify the P‐ÂQRS starting with a negative lead. We can first check whether an external lead (aVL or III) is negative or isodiphasic; if more than one lead is negative, all of these leads can be checked individually until a positive or isodiphasic lead is found. For example, if lead III is isodiphasic and lead aVF is positive, the P‐ÂQRS is 120° (the other measurement, −60°, is disregarded). If lead III is negative and lead aVF is positive, the P‐ÂQRS is located in the middle of them, that is, at 105°. If lead aVF is isodiphasic, the P‐ÂQRS is 90°. If it is negative, lead II is examined. The rest of the leads can be evaluated in the same way.

**TABLE 1:** The morphology of limb leads and the P‐ÂQRS, ÂQRS

A	III	aVF	II	aVR	I	aVL	P‐ÂQRS	ÂQRS
	→	↑	↑	↓	↑	↑	120	30
	↓	↑	↑	↓	↑	↑	105	15
	↓	→	↑	↓	↑	↑	90	0
	↓	↓	↑	↓	↑	↑	75	−15
	↓	↓	→	↓	↑	↑	60	−30
	↓	↓	↓	↓	↑	↑	45	−45
	↓	↓	↓	→	↑	↑	30	−60
	↓	↓	↓	↑	↑	↑	15	−75
	↓	↓	↓	↑	→	↑	0	−90

B	aVL	I	aVR	II	aVF	III	P‐ÂQRS	ÂQRS
	→	↑	↓	↑	↑	↑	−30	60
	↓	↑	↓	↑	↑	↑	−15	75
	↓	→	↓	↑	↑	↑	0	90
	↓	↓	↓	↑	↑	↑	15	105
	↓	↓	→	↑	↑	↑	30	120
	↓	↓	↑	↑	↑	↑	45	135
	↓	↓	↑	→	↑	↑	60	150

Abbreviations: ÂQRS, the mean QRS axis; P‐ÂQRS, The axis perpendicular to the mean QRS axis; →, isodiphasic; ↑, positive; ↓, negative; A, the lead is not positive from lead III; B, the lead is not positive from aVL.

Once the P‐ÂQRS is determined, it is easy to determine the ÂQRS. We approached determining ÂQRS in the following way:

1. Check lead aVL and lead III for isodiphasic or negative qualities. If both are positive, the ÂQRS is 45°; if both are negative, the ÂQRS is −135°.
2. If lead III is isodiphasic or negative, the degree of the P‐ÂQRS minus 90° is the degree of the ÂQRS.
3. If lead aVL is isodiphasic or negative, the degree of the P‐ÂQRS plus 90° is the degree of the ÂQRS.

If lead aVL is isodiphasic, lead I is positive, the PP‐ÂQRS is −30° (the other measurement, 150°, is disregarded), and the ÂQRS is 60°. If lead aVL is negative, lead I is positive, the P‐ÂQRS is located in the middle—that is, at −15°, and the ÂQRS is 75°. If lead I is isodiphasic, the P‐ÂQRS is 0°, and the ÂQRS is 90°. If it is negative, lead aVR is examined. The rest of the leads can be evaluated in the same way.

Using this procedure, the ÂQRS may be calculated with a precision of 15°.

We investigated 200 recorded ECGs from the routine daily work of ECG technicians who used electrocardiographs in July 2019 at the First People's Hospital of Jingmen. Uncertain electric axes and bundle branch blocks were excluded. Two blinded observers made the measurements independently with the P‐ÂQRS method and the traditional bipolar method, we obtained the mean QRS axis from standard leads I and III, and we plotted the results on a hexaxial reference frame. The results were compared with each other and with computer interpretations. Some ECGs of bundle branch blocks were analyzed separately. The study was approved by the First People's Hospital of Jingmen Ethics Committee.

Statistical analysis was performed with SPSS 21.0, including analysis of each method, producing mean and SDs. Correlation between each of the methods was completed by using the Pearson correlation coefficient. *P* < .05 was regarded as statistically significant. Bland‐Altman method comparison was performed with GraphPad Prism 8 between the P‐ÂQRS method and bipolar method.

## 3 RESULTS

**FIGURE 2:**
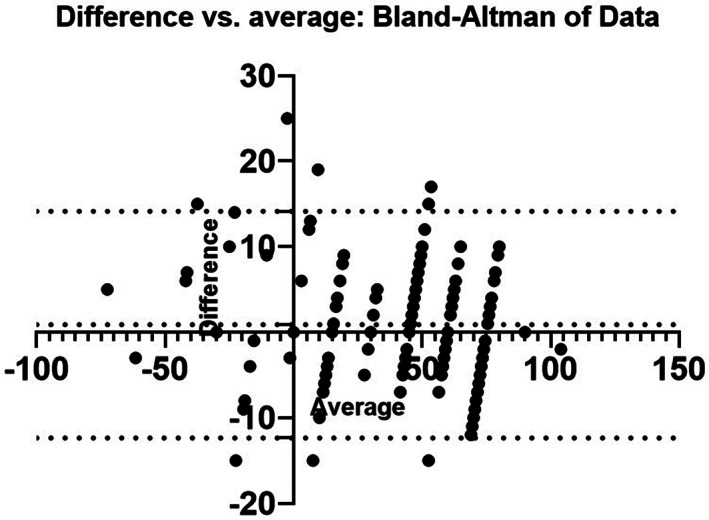
The Bland‐Altman plot of differences between the P‐ÂQRS and bipolar methods shows the mean bias ±SD (0.885 ± 6.761) and the limits of agreement (−12.37 and 14.14)

We reviewed the standard 12 lead electrocardiograms of 200 patients. The mean and SDs were remarkably similar (bipolar: 412.94 ± 34.13, P‐ÂQRS: 41.25 ± 34.38, computer axis: 42.935 ± 32.23). The Pearson correlation coefficients for each method were listed in Table 2. The correlation coefficient between the P‐ÂQRS method and the bipolar method was 0.976 (*P* < .001). The correlation coefficient between the bipolar method and the computer calculations was 0.931 (*P* < 0.001). The maximum difference between the P‐ÂQRS method and the bipolar method was 25°, and the maximum difference between the computer calculations and the bipolar method was 69°. The Bland‐Altman plot showed the mean bias ± SD between the P‐ÂQRS method and the bipolar method as 0.885 ± 6.761, and the limits of agreement were −12.37 and 14.14 (Figure 2).

**TABLE 2:** Correlations among the methods

Method	Bipolar	P‐ÂQRS	Computer axis
Bipolar	1	0.976	0.931
P‐ÂQRS	0.976	1	0.941
Computer axis	0.931	0.941	1

**FIGURE 3:**
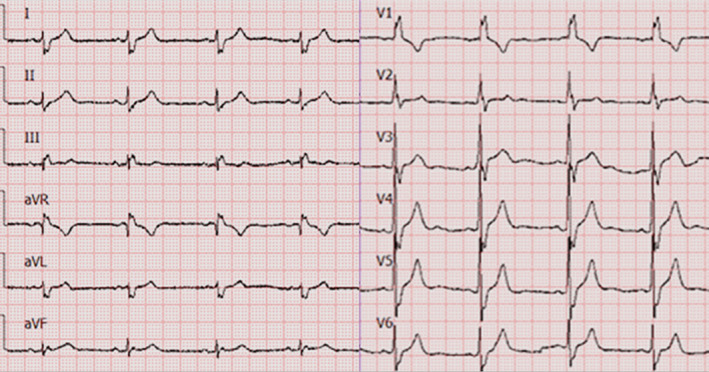
An ECG of a right bundle branch block, lead aVL is negative, lead I is negative, lead aVR is isodiphasic with a wide R‐wave

In the analysis of the ÂQRS, both amplitude and area require consideration, but the latter is difficult to calculate. Thus far, all methods for determining the ECG axis do not consider area, which, in most cases, can be disregarded. However, some leads appear as wide Q, S, or R waves, therefore requiring the examination of area in some cases. Consequently, we analyzed 40 bundle branch blocks ECGs separately (20 ECGs of right bundle branch block and 20 ECGs of left bundle branch block). For one right bundle branch block ECG, the P‐ÂQRS method generated values that differed by 28° from those determined via the bipolar approach (Figure 3). The ÂQRS was 148°, as identified through the bipolar method, and 120°, as identified by using the P‐ÂQRS approach. If only amplitude is considered, lead aVR is isodiphasic, and the ÂQRS is 120°. If area is examined, the R wave in lead aVR is wide, lead aVR is positive, and the ÂQRS is 135°.

## 4 DISCUSSION

Experts have recommended a variety of methods for determining the ÂQRS, however, no individual method is ideal.^5^ In most cases, an electrocardiograph can automatically and accurately analyze ECG axes, but mistakes are occasionally possible. Our analysis revealed that 2 out of 200 ECGs have a difference of more than 60°. Textbooks recommended determining the ÂQRS using the waveforms of leads I, II, and III.^6^, ^7^ They summarized the ÂQRS degrees for a variety of QRS configurations, for example, if leads I and II are positive, and III is negative, the ÂQRS is 0. When using this method, one must remember the degree of the ÂQRS for various waveforms, and these measurements are only accurate to within 30°. This method is similar to our method; however, we provided a faster determination.

As anticipated, our study indicated all three methods showed excellent agreement. The bipolar approach is considered to be the most reliable, the correlation coefficient of the bipolar approach and the P‐ÂQRS technique is 0.976. In the majority of situations, the disparity between the two methods is 0° to 15°, but in a few cases, the disparity is 15° to 25°.

The Bland‐Altman comparison method demonstrates the consistency of the bipolar and P‐ÂQRS methods. The two ECGs with the most significant differences had a wide S‐wave in lead III. When identifying the electric axis, waveform area must be considered in addition to amplitude; however, measuring the waveform area using the bipolar method proved difficult, so we opted to measure the ÂQRS with leads I and II, which reduced the difference significantly. The P‐ÂQRS of one ECG was −15°, 10° using the bipolar method and when measured with leads I and II, the ÂQRS was −18°, differing by 28° between lead combinations. A previous study reported that ÂQRS may differ by up to 35° using different lead combinations,^8^ suggesting that the bipolar method is not always reliable. The effect of a wide S‐wave in the lead around the P‐ÂQRS must also be considered; in this case we ought to increase the weight of the wide waveform which eliminates the 15° discrepancy in two ECGs.

So far, there is no method to accurately reflect the actual electric axis. The bipolar method is largely reliable, but it also presents errors when determining the electric axis.^8^ Because the bipolar method uses the amplitude of lead I and III to determine the electric axis, if such amplitude is affected locally under pathological conditions, the electric axis may be inaccurate. The P‐ÂQRS method is unaffected by the local potential, which can ensure that the result is not too different from the actual axis. Theoretically, the difference between the P‐ÂQRS method and the actual electric axis approach should be within 0° to 10°. It does not usually compel a very accurate QRS axis in clinical conditions, and an error within 10° is completely acceptable.

The isodiphasic lead method was quite popular, allowing the ÂQRS to be determined within 10 seconds by an experienced doctor; however, our new method may be more convenient. The first step of the old method is to search for an isodiphasic lead among the six limb leads. Once such a lead is identified, the QRS axis is perpendicular to this lead, since the perpendicular line has two degrees, while another lead needs to be checked in order to determine the QRS axis. Because most of the ECG axis is normal or only slightly deviated, when using our new method, the ÂQRS can be determined after checking only two or three leads. In addition, if there is no isodiphasic lead, then clinicians spend a few extra seconds determining whether such a lead exists.

### 4.1 Limitations

Only 240 ECGs were analyzed; however, the utilization of the P‐ÂQRS to determine the ÂQRS is a mature method that does not need widespread testing.

### 4.2 Summary

At present, there are numerous methods for analyzing the QRS axis, none of which is perfect. We used the new method to quickly and accurately determine the QRS axis—this method is easier to navigate and is worthy of clinical application.

## CONFLICT OF INTEREST

The authors declare no potential conflict of interests.

## Data Availability

Some or all data, models, or code generated or used during the study are available from the corresponding author by request.

## References

[1] Laiken S , Laiken N , O'Rourke RA , Karliner JS . A rapid method for frontal plane axis determination in scalar electrocardiograms. Am Heart J. 1973;85(5):620‐623. 4266821 10.1016/0002-8703(73)90167-1

[2] Martinez‐Diaz J , Escabi‐Medoza J , Oviedo R , Acevedo J . Quick method for mean frontal QRS axis determination. Bol Asoc Med P R. 2008;4:19‐24. 19400525

[3] Grant RP . Clinical electrocardiography. New York, NY: McGraw‐Hill Book Company; 1957.

[4] Pahlm US , O'Brien JE , Pettersson J , et al. Comparison of teaching the basic electrocardiographic concept of frontal plane QRS axis using the classical versus the orderly electrocardiogram limb lead displays. Am Heart J. 1997;134(6):1014‐1018. 10.1016/s0002-8703(97)70020-6. 9424060

[5] Spodick DH , Frisella M , Apiyassawat S . QRS axis validation in clinical electrocardiography. Am J Cardiol. 2008 Jan;101(2):268‐269. 18178420 10.1016/j.amjcard.2007.07.069

[6] de Luna AB , Goldwasser D , Fiol M , Bayés‐Genis A . Surface electrocardiography In: Fuster V, Harrington RA, Narula J, Eapen ZJ, eds. Hurst's The Heart. 14th ed. New York, NY: McGrawHill; 2017.

[7] Bayés de Luna A, ed. Characteristics of the normal electrocardiogram: normal ECG waves and intervals Clinical electrocardiography. 4th ed. Chichester, UK: Wiley‐Blackwell Publishing; 2012:67‐94.

[8] Okamoto N , Kaneko K , Simonson E , Schmitt OH . Reliability of individual frontal plane axis determination. Circulation. 1971;44(2):213‐219. 10.1161/01.cir.44.2.213. 5562557

